# Scalable and Recyclable All‐Organic Colloidal Cascade Catalysts

**DOI:** 10.1002/anie.202008104

**Published:** 2020-10-28

**Authors:** Chen Chen, Nicole Janoszka, Chin Ken Wong, Christian Gramse, Ralf Weberskirch, André H. Gröschel

**Affiliations:** ^1^ Physical Chemistry University of Münster Corrensstraße 28–30 48149 Münster Germany; ^2^ Faculty of Chemistry and Chemical Biology TU Dortmund University Otto-Hahn-Straße 6 44227 Dortmund Germany

**Keywords:** cascade reactions, catalysis, compartmentalization, emulsion polymerization, microreactors

## Abstract

We report on the synthesis of core–shell microparticles (CSMs) with an acid catalyst in the core and a base catalyst in the shell by surfactant‐free emulsion polymerization (SFEP). The organocatalytic monomers were separately copolymerized in three synthetic steps allowing the spatial separation of incompatible acid and base catalysts within the CSMs. Importantly, a protected and thermo‐decomposable sulfonate monomer was used as acid source to circumvent the neutralization of the base catalyst during shell formation, which was key to obtain stable, catalytically active CSMs. The catalysts showed excellent performance in an established one‐pot model cascade reaction in various solvents (including water), which involved an acid‐catalyzed deacetalization followed by a base‐catalyzed Knoevenagel condensation. The CSMs are easily recycled, modified, and their synthesis is scalable, making them promising candidates for organocatalytic applications.

## Graphical abstract

An all‐organic core–shell colloidal catalyst was synthesized through two‐step surfactant‐free emulsion polymerization. The acid/base colloidal catalyst shows high catalytic activity in water, can be scaled up and recycled, indicating its potential for practical applications.

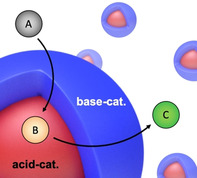

Cascade reactions that occur consecutively in one‐pot have attracted increasing attention in the last few decades due to their simplified preparation, shortened overall reaction times, and the reduced amount of required solvent (less purification steps).[^1^, ^2^, ^3^, ^4^, ^5^] The main challenge for conducting multistep catalytic reactions in one pot is to provide suitable conditions that allow to combine incompatible catalysts without their mutual interference or quenching, for example, acid and base catalysts would neutralize each other.[^6^, ^7^] In living cells, one key concept for solving this problem is compartmentalization, that is, the spatial separation of catalysts in nanodomains of organelles. This spatial separation prevents cross‐reactions between the catalysts (and substrates) and they are only able to participate in specific steps of the reaction to give the desired product.^[8]^

Inspired by catalyst site‐isolation in nature, researchers have adapted this concept to separately store catalysts in different containers^[9]^ including linear polymers,^[10]^ polymer‐based nanostructures (e.g. micelles,^[11]^ star polymers,^[12]^ bottlebrushes,^[13]^ polymersomes,[^14^, ^15^, ^16^] hydrogels^[17]^), silica particles (e.g. mesoporous,[^18^, ^19^] double shell,^[20]^ yolk‐shell^[21]^), graphene oxide,^[22]^ and Pickering emulsions.[^23^, ^24^] An early elegant example is given by two star polymers each containing one acid and one base catalyst for the deacetalization‐Henry cascade reaction in one‐pot in DMF.^[6]^ To reduce the diffusion path of reactants from one catalyst to the next and thus, potentially lowering reaction time, more recent efforts are directed towards combining both catalysts in the same particle in only few nanometer distance.[^25^, ^26^, ^27^] For instance, self‐assembled and cross‐linked micelles that carry an acid catalyst in the corona and a base catalyst in the micelle core demonstrated high catalytic activity in organic solvents^[28]^ and even in water.^[26]^

Although these excellent examples have achieved to combine incompatible acid/base catalysts in one container and showed successful cascadic reactions, there are still limitations to practical application in part due to multistep synthetic procedures or special components that prevent their synthesis in large quantities, as well as challenges in separation of catalysts from the reaction mixture limiting reuse capabilities. Based on these challenges, it would be desirable to explore alternative particle systems for cascade catalysis that are easy to scale‐up, recycle, and modify, as these are relevant requirements for practical applications. In that regard, core–shell microparticles (CSMs) made by established two‐step surfactant‐free emulsion polymerization^[29]^ are promising candidates as they can be synthesized from a variety of monomers (chemically different nanodomains may contain different functional monomers), they can be produced in large quantities, and are removed from the reaction mixture by straightforward sedimentation or centrifugation. Despite these beneficial properties, CSMs have not been explored as colloidal cascade catalysts so far.

Here, we report on the preparation of organic CSMs that perform an acid‐catalyzed deacetalization and a base‐catalyzed Knoevenagel condensation in a one‐pot cascade reaction. Paying attention to the preparation conditions, the CSMs are prepared in three scalable (up to 100 g L^−1^) synthetic steps, and can be recycled multiple times with minimal variation in catalytic activity. The synthetic procedure is outlined in Scheme 1.

**Scheme 1:**
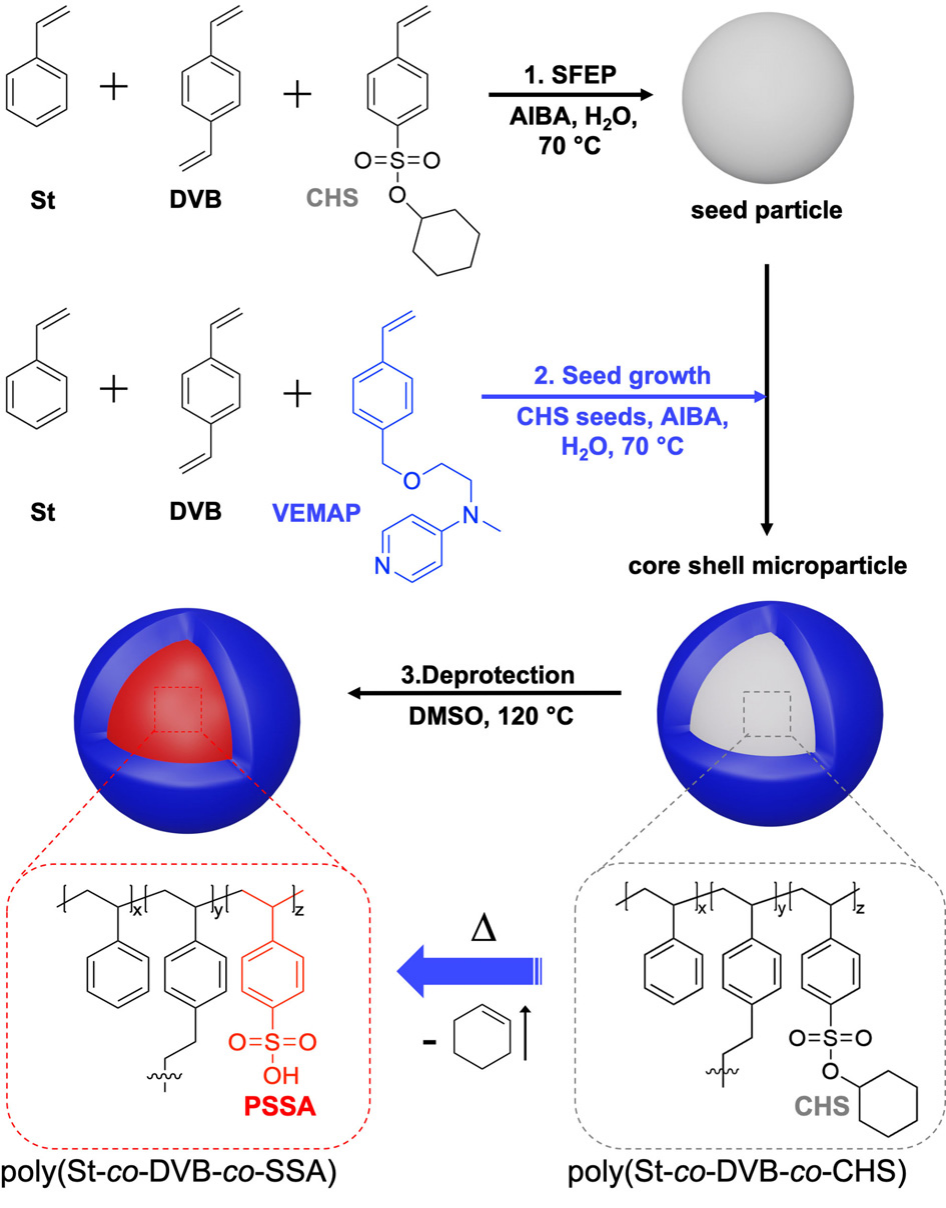
Scheme of the synthetic procedure of the all‐organic core–shell acid/base particle.

In brief, we first synthesized cross‐linked polystyrene (PS) “seeds” bearing the protected acid functionality. For that, we copolymerized seed particles from (i) styrene (St), (ii) divinylbenzene (DVB), and (iii) cyclohexyl‐*para*‐styrenesulfonate (CHS) using surfactant‐free emulsion polymerization (for details see SI). PS forms the hydrophobic compartment, DVB serves as cross‐linker, and CHS is the hydrophobic precursor of the acid catalyst. The CHS monomer was synthesized according to literature.[^30^, ^31^] In the second step, we used seeded emulsion polymerization to form a shell on the CHS seeds consisting of St, DVB and the base catalyst, 4‐*N*‐(4‐vinylbenzyl)oxyethyl‐*N*‐methylamino‐pyridine (VEMAP). The VEMAP synthesis was adapted from previous reports involving two synthetic steps.[^32^, ^33^] After completion of the shell, the CHS core can be readily deprotected via thermal treatment at 120 °C to yield catalytically active polystyrene sulfonic acid (PSSA) (see Supporting Information for details).^[31]^ Both the SSA and VEMAP content in the CSMs were determined by acid‐base titration, which gave 0.034 mmol of SSA and 0.094 mmol VEMAP per gram CSMs (Figure S5 & S6).

The CHS seeds and (CHS‐deprotected) SSA/VEMAP CSMs were characterized by TEM (Figure 1 A,B). Image analysis showed that the CHS seeds had an average diameter (*d*_TEM_) of 465±30 nm that increased to 774±50 nm for the SSA/VEMAP CSMs. The lack of electron contrast made it difficult to discern the SSA core from the VEMAP shell in the TEM images. In order to confirm that the SSA/VEMAP particles indeed have a core–shell structure, we selectively labelled the shell with a fluorescent dye (fluorescein) during shell formation and imaged the CSMs by confocal laser scanning microscopy (CLSM, see SI for labelling procedure). As can be seen from the inset in Figure 1 B, only the shell is visible in CLSM (green), confirming the core–shell structure of the particles. We also imaged the CHS seeds and SSA/VEMAP CSMs by SEM (Figure 1 C,D). Both set of samples form ordered close‐sphere packing after sample preparation via drop‐casting, suggesting high uniformity in size before and after shell formation. DLS measurements confirmed an increase of hydrodynamic diameter from *d*_h_=486±164 nm for the seeds to *d*_h_=772±75 nm for the SSA/VEMAP CSMs in line with our TEM/SEM observations. Both species exhibit narrow size distributions (PDI of CHS seeds and SSA/VEMAP CSMs are 0.14 and 0.08, respectively), where the higher PDI value of CHS seeds was attributed to slight aggregation after one day (Figure S9).

**Figure 1:**
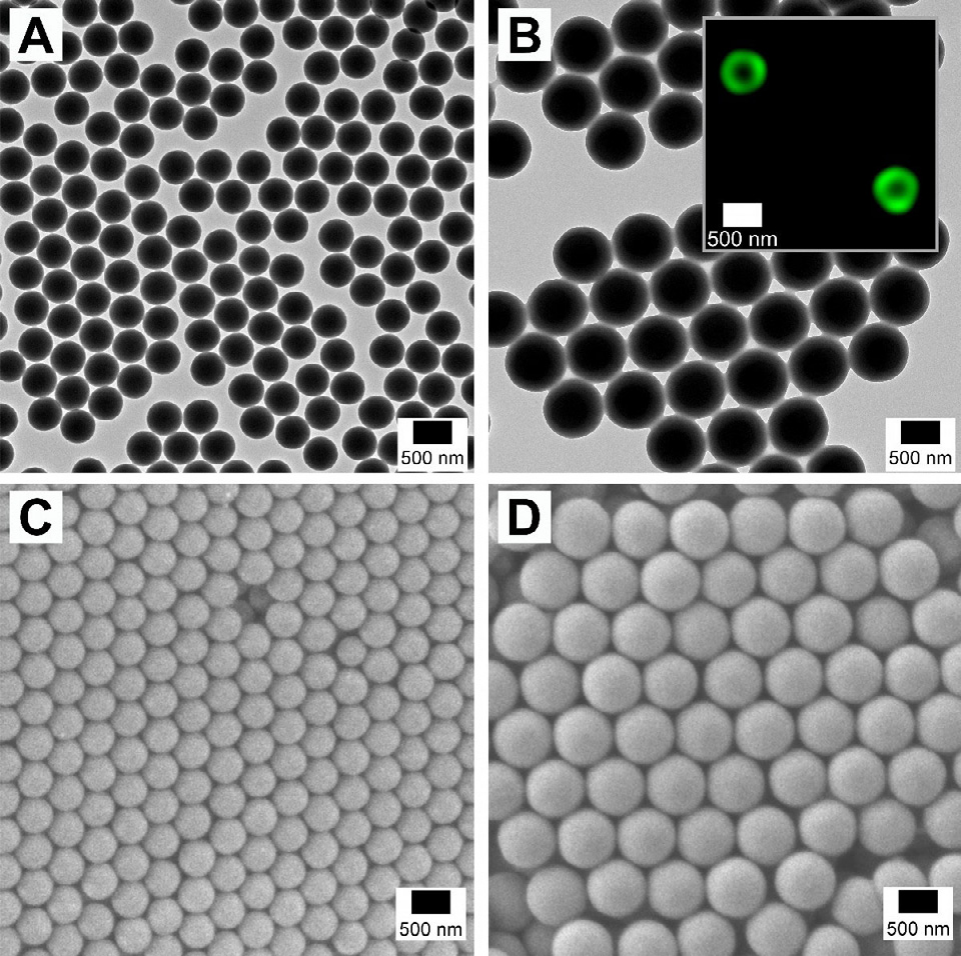
TEM images of (A) CHS seeds and (B) SSA/VEMAP core–shell particles. Inset in B shows a confocal image of core–shell particles with a shell structure that has been labelled with a fluorescent dye, fluorescein. SEM images of (C) CHS seeds and (D) SSA/VEMAP core–shell particles.

Although the synthetic steps towards stable CSMs carrying both acid and base seemed simple, they required several considerations. First, the CHS monomer (acid catalyst source) incorporated in the core must be polymerized in its protected form to avoid neutralization with VEMAP during the second polymerization. Protective groups cannot be removed by acids or bases as this would likewise result in deactivation of either of the catalysts, leaving thermal deprotection as one of the viable options. Second, 2,2′‐azobis(2‐methylpropionamide) dihydrochloride (AIBA) with positive charge was selected to initiate both polymerizations, because potassium persulfate (KPS) with negative charge merely led to precipitation after St/DVB/VEMAP shell formation (although St/DVB/CHS seeds were monodisperse and stable) (Figure S7). The AIBA dosage for each step should be also precisely controlled to prevent destabilization and aggregation of CSMs during emulsion polymerization. We attributed both aggregation effects to slow concurrent hydrolysis of CHS, which provides negative charges on the particle surface and caused undesired coagulation during the emulsion polymerization. We found it crucial to follow the above synthetic order (first CHS seeds, then VEMAP shell) (Figure S7) and to limit the polymerization time in the first step to a maximum of 4 h. Longer polymerization times led to higher monomer conversion, but likewise to destabilization of the seeds (Figure S8). The issues mentioned above can be overcome by reducing the amount of CHS monomer, allowing for longer polymerization times. However, this will inevitably lead to lower catalytic activity due to the lower PSSA content.

Next, we investigated the catalytic performance of the CSMs in the one‐pot cascade deacetalization and Knoevenagel reaction (reaction scheme in Table 1). We first tested the reaction in a mixture of DMSO/H_2_O (40:1 v/v), as is typically used in the literature.^[13]^ The cascade reaction was initiated by adding 1 mol eq. of the benzaldehyde dimethyl acetal **1** (starting reagent) and 1 mol eq. of ethyl cyanoacetate **2 b** (second reagent required for the second step) into a dispersion containing 0.01 mol eq. of CSMs, and heated the reaction mixture to 90 °C. After 24 h, the conversion was determined by NMR spectroscopy using methanol*‐d4* as solvent. Only trace amounts of the starting reagent **1** and 96 % yield of benzylidene ethyl cyanoacetate **3** (final product) were found underlining the excellent catalytic activity of SSA/VEMAP CSMs (entry *a* in Table 1). As will be discussed later the reaction can be improved further to reach full conversion to **3** within only 2 h.

**Table 1:** Cascade deacetalization–Knoevenagel reaction. 
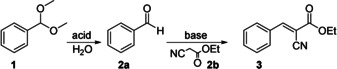

Entry	Catalyst	Compounds	Conv. **1**	Yield **2 a**	Yield **3**
*a*	SSA/VEMAP^[a]^	**1**–**3**	>99	4	96
*b*	None^[a]^	**1**–**3**	6	4	2
*c*	None^[b]^	**2**–**3**	–	–	28
*d*	CHS/VEMAP^[a]^	**1**–**3**	7	tr	7
*e*	M_SSA_ + M_VEMAP_^[a]^	**1**–**3**	8	8	tr
*f*	M_SSA_ + SSA/VEMAP^[a]^	**1**–**3**	>99	98	2
*g*	M_VEMAP_ + SSA/VEMAP^[a]^	**1**–**3**	15	2	13
*h*	SSA/VEMAP^[c]^	**1**–**3**	>99	tr	>99
*i*	None^[c]^	**1**–**3**	62	32	30

Reaction conditions: [a] benzaldehyde dimethyl acetal, **1** (1 mol eq.), ethyl cyanoacetate, **2 b** (1 mol eq.), catalysts (0.0038 mol eq. SSA, 0.01 mol eq. VEMAP), DMSO/H_2_O (40:1 v/v), 90 °C, 24 h; [b] benzaldehyde **2 a** introduced directly as the starting reagent; [c] benzaldehyde dimethyl acetal, **1** (1 mol eq.), ethyl cyanoacetate, **2 b** (1 mol eq.), catalyst (0.0038 mol eq. SSA, 0.01 mol eq. VEMAP), H_2_O, 90 °C, 2 h. tr = trace.

In order to confirm that the CSMs did effectively catalyze the cascade reaction as per entry *a* in Table 1, we performed several negative control experiments. First, we attempted the cascade reaction without any catalytic particles (Entry *b* in Table 1). As expected, this led to only minimal conversion of the starting reagent **1** into the intermediate product **2 a** (benzaldehyde), and little formation of the final product **3**. Next, we tested only the Knoevenagel reaction (i.e., the second catalytic reaction) in the absence of any catalytic particles (Entry *c* in Table 1). Under this condition, the yield of the final product **3** was measured to be 28 %, which indicates that some background conversion of **2 a** to **3** occurs even in DMSO/H_2_O. Despite this, the yield of **3** is about 3.5‐fold lower as compared to the CSMs. We can therefore confidently assume that the SSA/VEMAP CSMs did perform the cascade reaction. We further tested the cascade reaction using CSMs without deprotection of the acid in the core (entry *d* in Table 1). In this case, only a small amount (7 %) of starting reagent **1** was found to have hydrolyzed into the intermediate product **2 a**. This was expected as this system lacks the acid catalyst, SSA, which is necessary to form the intermediate product **2 a**. Note, however, that we were only able to detect trace amounts **2 a** in the reaction mixture as the intermediate product **2 a** is rapidly consumed in the second catalytic reaction to give the final product **3** due to the presence of VEMAP. Expectedly, we measured an equimolar yield (7 %) of final product **3**. Having confirmed the activity of CSMs, we conducted several reference experiments with molecular catalysts to determine the influence of catalyst neutralization. We first carried out the cascade reaction with SSA and VEMAP monomers (M_SSA_ and M_VEMAP_) (Entry *e* in Table 1). Only minimal conversion of starting reagent **1** into intermediate product **2 a** and trace amounts of final product **3** were found, which confirms that mutual neutralization between acid/base monomers eliminated most of the active species. Mixing SSA monomer (M_SSA_) and SSA/VEMAP CSMs (Entry *f* in Table 1) gave almost full conversion of starting reagent **1** into intermediate product **2 a** (as expected from excess SSA), but only low conversion to the final product **3**, which is attributed to the lack of base catalyst (SSA monomer neutralized VEMAP in the CSMs). Mixing VEMAP monomer (M_VEMAP_) and SSA/VEMAP CSMs (Entry *g* in Table 1), resulted in only a low conversion of the starting reagent **1** to intermediate product **2 a** (15 %) due to quenching of SSA in the CSMs through M_VEMAP_. Since there is now an excess of VEMAP catalyst, **2 a** was in turn converted to the final product **3** in high yields (13 %).

To investigate the advantages of the core–shell structure, we prepared microparticles where the acid (SSA/St) and base (VEMAP/St) catalyst are separated from each other in individual microparticles (see SI for experimental procedures). We mixed both microparticles and carried out the cascade reaction to compare their reaction kinetics with CSMs (at same catalyst concentrations). Surprisingly, the separate microparticles were also able to catalyze the cascade reaction, albeit at a lower catalytic activity (Figure S11 & S12B). Unlike the CSMs which catalyzed the cascade reaction to near‐full conversion (92 %) in 6 h, the mixture of individual particles only achieved 45 % conversion in the same time period, which confirms the importance of the core–shell morphology. As illustrated in Figure 2 A, the diffusion path between core and shell is short (unlike interparticle diffusion), which allows for efficient conversion of intermediate products into the final product. We attribute the still decent reaction rate of the separate microparticles to strong agglomeration that brings acid and base microparticles in close contact (Figure S12A). We believe that the cascade reaction would be even slower if both microparticles remained stable and were thus not in contact to exchange reactants. According to the kinetics plots (Figure S11), the reaction times of the two catalytic steps are largely different (acid‐catalyzed deacetalization 30 min, base‐catalyzed Knoevenagel condensation 360 min), which further corroborates the relevance of an acid‐core and a base‐shell. Using this composition, the formed intermediate **2 a** must pass the base catalyst in the shell ensuring its conversion (if the acid was in the shell, the more hydrophilic **2 a** could diffuse into the continuous phase without conversion). Further, the retention time of the **2 a** within the CSM is increased, additionally enhancing the reaction rate.

**Figure 2:**
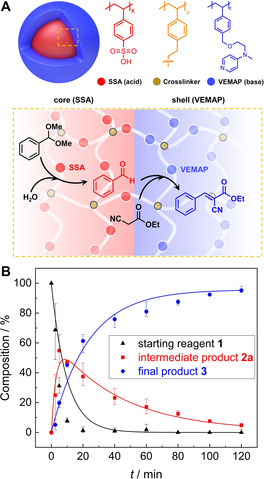
(A) Scheme of the deacetalization–Knoevenagel cascade reaction route in the CSM. (B) Reaction kinetics of SSA/VEMA CSMs in water followed over time in NMR.

Having verified the catalytic activity of the SSA/VEMAP CSMs in DMSO/H_2_O (40:1 v/v), we explored their performance in pure water as opposed to solvent mixtures (as often used in other catalyst systems[^12^, ^13^, ^28^]) to demonstrate that our system can also perform in a “green” manner. The SSA/VEMAP CSMs performed very well in pure H_2_O leading to full conversion of **3** in 2 h (Entry *h* in Table 1). As shown in the Figure 2 B, we monitored the conversion of this reaction by plotting the consumption of the starting reagent **1** into the intermediate product **2 a**, as well as the formation of the final product **3**. For that, we drew aliquots from the reaction mixture at predefined times and monitored the conversion with NMR in methanol*‐d4* (see Figure S13 for calculation of conversions). According to the kinetics plot (Figure 2 B), the amount of starting reagent **1** decreased exponentially right after the start (black trace). Within 10 min, ≈95 % of **1** was consumed as a result of the deacetalization reaction, which was facilitated by the SSA core. We attribute this accelerated reaction rate to enrichment of **1** and **2 b** within the hydrophobic CSMs due to their low solubility in H_2_O. Whereas both organic substrates homogeneously distribute in organic solvents (e.g. DMSO) in the entire volume on the reaction mixtures, they preferentially accumulated within the CSMs in H_2_O, which raised the substrate concentration in the vicinity of the catalysts and resulted in faster reaction rate.^[26]^ We note that the deacetalization, which is in essence a hydrolysis reaction, is likely enhanced by the presence of the large excess of H_2_O (Table 1 entry *i*, cascade reaction without catalysts in water). Accordingly, within the same period of time (10 min), the amount of intermediate product **2 a** increased (red trace). However, after 10 min of reaction time, we recorded an exponential decrease of **2 a** due to its conversion into **3**, which steadily increased over time until it began to plateau as the starting reagent **1** and intermediate product **2 a** were nearing depletion.

Recyclability is an important property for catalysts^[20]^ that should be easy to remove from the reaction mixture for reuse and to give pure products. For that, SSA/VEMAP CSMs were centrifuged and redispersed several times to catalyze another batch. As shown in Figure 3 A, we monitored the catalytic performance of the SSA/VEMAP CSMs over six reaction cycles, where **1** was still converted with 97 % yield giving product **3** in 95 % yield. DLS measurements confirmed the stability of the CSMs after each recycling step. The particles maintained a narrow monomodal size distribution, which indicated that no aggregation occurred during catalysis or recycling (Figure 3 B). SEM analysis also showed no obvious change to the CSMs structure after the cascade reaction (Figure S14).

**Figure 3:**
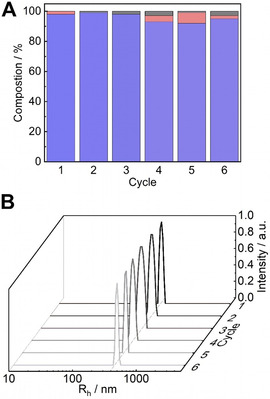
(A) Compositions of starting reagent **1** (black), intermediate product **2 a** (red) and final product **3** (blue) of six reaction cycles catalysed by recycled SSA/VEMAP core–shell particles and (B) the corresponding size distribution after each cycle.

In summary, we demonstrated the synthesis of narrowly dispersed, all‐organic colloidal catalysts via two‐step surfactant‐free emulsion polymerization with control over chemical composition and site‐isolation of the catalysts. Specifically, we developed a synthetic strategy to selectively anchor acid (SSA) and base (VEMAP) catalysts to the core and shell of CSMs without mutual quenching. The spatial separation of both catalysts was confirmed in CLSM. These CSMs were readily used in a one‐pot acid/base‐catalyzed deacetalization–Knoevenagel cascade reaction, and showed excellent activity and recyclability. Emulsion polymerization has high scale up potential as it is widely used in industry for decades to make colloidal microparticles. The herein developed colloidal cascade catalyst were synthesized on a 25 g scale (Figure S15) and the corresponding cascade reaction could be extended to synthesize the final product **3** on the 10 g scale with still excellent yields (Figure S16). Currently, we explore routes to extend this strategy to multicompartment microparticles with 20 nm domain spacing, more complex reactions beyond this model system, and particle geometries that might offer a general way for simple and green organic synthesis with high efficiencies.

## Conflict of interest

The authors declare no conflict of interest.

## Supporting information

As a service to our authors and readers, this journal provides supporting information supplied by the authors. Such materials are peer reviewed and may be re‐organized for online delivery, but are not copy‐edited or typeset. Technical support issues arising from supporting information (other than missing files) should be addressed to the authors.

Supplementary
