# Supplementary material for: Scalable and Recyclable All‐Organic Colloidal Cascade Catalysts
Source: Angew Chem Int Ed Engl. 2020 Oct 28;60(1):237–41. doi: 10.1002/anie.202008104 (PMC7821152; doi:10.1002/anie.202008104)
Supplement: Supplementary file 1 — Supplementary [file ANIE-60-237-s001.pdf]

Angewandte Chemie
Eine Zeitschrift der Gesellschaft Deutscher Chemiker

Supporting Information

Scalable and Recyclable All-Organic Colloidal Cascade Catalysts

*Chen Chen, Nicole Janoszka, Chin Ken Wong, Christian Gramse, Ralf Weberskirch, and André H. Gröschel**

anie_202008104_sm_miscellaneous_information.pdf

Author Contributions

C.C. Investigation: Lead; Visualization: Lead; Writing - Original Draft: Equal; Writing - Review & Editing: Equal
N.J. Investigation: Supporting; Validation: Supporting; Writing - Review & Editing: Supporting
C.W. Validation: Supporting; Writing - Original Draft: Supporting; Writing - Review & Editing: Supporting
C.G. Investigation: Supporting; Validation: Supporting; Writing - Review & Editing: Supporting
R.W. Supervision: Supporting; Writing - Review & Editing: Supporting
A.G. Conceptualization: Lead; Funding acquisition: Lead; Supervision: Lead; Validation: Lead; Writing - Original Draft: Supporting; Writing - Review & Editing: Lead.

Table of Contents

Table of Contents **1**
Experimental Procedures **2**
Materials 2
Measurements 2
Synthesis of *para*-styrene sulfonyl chloride (*p*SSC) 2
Synthesis of cyclohexyl-*para*-styrene sulfonate (CHS) 2
Synthesis of 4-(*N*-methyl-*N*-(2-hydroxyethyl)amino)pyridine (HEMAP) 2
Synthesis of 4-*N*-(4-vinylbenzyl)oxyethyl-*N*-methylaminopyridine (VEMAP) 2
Preparation of CHS seed particles (core) and VEMAP particles 2
Preparation of CHS/VEMAP CSMs 3
Deprotection of CHS/VEMAP CSMs 3
Catalyst content test of SSA/VEMAP CSMs 3
Catalytic performance test of SSA/VEMAP CSMs 3
Supporting Figures **4**
Synthesis of the CHS monomer 4
Synthesis of the VEMAP monomer 5
Co-polymerization of St and CHS and the thermo-deprotection of the CHS/St particles 6
Co-polymerization of St and VEMAP 7
SSA content test of the CSMs *via* titration. 8
VEMAP content test of the CSMs *via* titration. 9
Influence of initiator for the polymerization of CSMs. 10
Influence of polymerization time of CHS seed 11
Dynamic light scattering measurements 12
The CLSM overview image of the CSMs. 13
Reaction kinetics of CSMs in DMSO/H_2O (40:1 V/V). 14
Catalytic performance comparison of CSMs and mixture of individual particles in DMSO/H_2O (40:1 v/v). 15
Kinetics of cascade reaction 16
SEM image of CSMs after recycling 17
Scale-up potential of surfactant-free emulsion polymerization. 18
Scaled-up potential of cascade reaction. 19
Supporting References **20**
Author Contributions **20**

Experimental Procedures

Materials

All chemicals were used as received unless stated otherwise. Cyclohexyl styrene sulfonate (CHS) and 4-*N*-(4-vinylbenzyl)oxyethyl-*N*-methylaminopyridine (VEMAP) were synthesized according to published procedures.[1–3] Styrene (St, 99%), divinylbenzene (DVB), 2,2'-azobis(2-methylpropionamide) dihydrochloride (AIBA), 4-*tert*-butylbenzenecatechol (TBC), thionyl chloride, sodium styrene sulfonate (SSS), cyclohexanol, pyridine, *N*-methylethanolamine, sodium hydride (NaH, 60% dispersion in mineral oil) and 4-vinylbenzyl chloride (90%) were purchased from Sigma-Aldrich. 4-chloropyridine hydrochloride (97%) was obtained from Acros Organics. *N,N*-dimethylformamide (DMF, 99.9%), ethyl acetate (EtOAc, 99%) dichloromethane (DCM) and chloroform ($CHCl_3$) was purchased from VWR international and DMF was dried over activated molecular sieve before use. Deuterated chloroform ($CDCl_3$, 99%), deuterated methanol (methanol-*d4*) and deuterated dimethyl sulfoxide (DMSO-*d6*) were purchased from Cambridge Isotopes Laboratories, Inc. Benzaldehyde dimethyl acetal (BDA, 99%) and ethyl cyanoacetate (EC, 98%) was purchased from Alfa Aesar. 4% $NaHCO_3$ solution and 5 M H_2SO_4 solution were prepared by sodium hydrogen carbonate and sulfur acid (98%) from Carl Roth. The styrene monomer was passed through a short silica column to remove the inhibitor. Ultrapure water was obtained from a MilliQ® Integral Water Purification System and used for the preparation of particles *via* surfactant-free emulsion polymerization.

Measurements

Proton nuclear magnetic resonance (^1H NMR) spectra were recorded in either $CDCl_3$ or methanol-*d4* at 25 °C on a Spinsolve 60 benchtop NMR spectrometer (Magritek). Dynamic light scattering (DLS) was conducted on a LS spectrometer operated with a Nd:YAG solid state laser (max. 100 mW constant power output at λ = 660 nm). Samples were prepared at a concentration of 0.1 g/L and was passed through a PTFE filter (5 µm pore size) prior to analysis in cylindrical quartz cuvettes (diameter 10 mm). Three intensity-time autocorrelation functions were measured at a scattering angle of 90° with an acquisition time of 60 s. The recorded data was analyzed with LS spectrometer v. 63 software package. The transmission electron microscopy (TEM) measurements were performed on a JEOL JEM-1400 Plus TEM, operating at an accelerating voltage of 120 kV, a point resolution of 0.38 nm as well as a line resolution of 0.2 nm. Images were recorded with 16-bit 4096×4096 Pixel CMOS digital camera and processed FIJI open-source software package. For sample preparation, one drop of the diluted colloidal suspension (0.1 mg/mL) was deposited on a carbon-coated copper grid (200 mesh, Electron Microscopy Sciences) and excess solution was blotted away using filter paper after 30 s. The scanning electron microscopy (SEM) measurements were performed on a SEM equipped with in lens-, chamber- as well as energy-selective detectors for 16-bit image series acquisition with up to 40,000×50,000-pixel resolution. Samples for SEM measurements were prepared by placing a drop of diluted colloidal suspension (0.1 g/L) on a silica wafer. After 30 s, the solution was blotted with a paper and the wafer dried under ambient conditions for at least 12 h. Afterwards, a layer of 5 nm was sputtered on the samples using a Quorum PP3010T-Cryo chamber with integrated Q150T-Es high-end sputter coater and Pt-Cd target. Confocal laser scanning microscopy (CLSM) measurements were performed on a Leica SP8 microscope equipped with an Argon Ion laser. An excitation wavelength of 488 nm was used to obtain the confocal image. The sample was prepared by placing a drop of diluted colloidal suspension (0.1 g/L) onto a glass cover slip for 30 s before blotting the drop away with a filter paper, and allowing the sample to dry overnight under ambient conditions.

Synthesis of *para*-styrene sulfonyl chloride (*p*SSC)

Dry DMF (56.64 g, 0.775 mol) and *tert*-butylbenzenecatechol (0.35 g, 2.1 mmol) were charged into a three-neck flask equipped with a condenser, drying tube, and stirred in an ice bath. Subsequently, thionyl chloride (82 g, 0.689 mol) was added dropwise into the mixture using a dropping funnel. After the complete addition of thionyl chloride, sodium styrene sulfonate (20.01 g, 96 mmol) was added as one portion into the flask. The mixture was allowed to stir for 3 h in an ice bath. After that, ice-cold water (300 mL) was added into the mixture and the mixture was extracted twice with chloroform. The combined organic phase was washed twice by pure water (200 mL) and dried over anhydrous Na_2SO_4. Chloroform was removed by rotary evaporator to yield the product *p*SSC as a yellow oil. The products was characterized by ^1H NMR (Figure S1A)[4].

Synthesis of cyclohexyl-*para*-styrene sulfonate (CHS)

Cyclohexanol (7.59 g, 75.8 mmol) and dry pyridine (75 g, 0.94 mol) were charged into a three-neck flask equipped with a condenser, drying tube, and stirred in an ice bath. Subsequently, *p*SSC was added dropwise into the mixture using dropping funnel. The slightly yellow mixture was vigorous stirring at room temperature for 16 h. Afterwards, the mixture was carefully mixed with 5 M sulfuric acid solution (187 mL) and subsequently extracted by chloroform. After washing twice by pure water (200 mL) and four times with 4% $NaHCO_3$ solution (4 × 150 mL), the organic phase was dried over anhydrous Na_2SO_4 and chloroform was removed by rotary evaporator. The crude product was purified by silica gel column chromatography using *n*-hexane/EtOAc (v/v, 9:1) as eluent to yield the product CHS as a clear liquid, as confirmed by ^1H NMR spectroscopy (Figure S1B).[4]

Synthesis of 4-(*N*-methyl-*N*-(2-hydroxyethyl)amino)pyridine (HEMAP)

4-(*N*-methyl-*N*-(2-hydroxyethyl)amino)pyridine was prepared according to the published literature procedure.[3] 4-Chloropyridine hydrochloride (22.5 g, 0.15 mol) was added into a flask equipped with a condenser containing 150 mL *N*-methylethanolamine. The mixture was then heated to 120 °C for 24 h. Excess *N*-methylethanolamine was removed by rotary evaporator as much as possible before appropriate amounts of 1M NaOH solution was added until a pH value of ~13 was achieved. The crude product was then extracted with DCM, the organic phase collected and dried over anhydrous Na_2SO_4. DCM was removed by rotary evaporator to yield HEMAP as an off-white solid, as confirmed by ^1H NMR spectroscopy (Figure S2A).

Synthesis of 4-*N*-(4-vinylbenzyl)oxyethyl-*N*-methylaminopyridine (VEMAP)

4-(*N*-methyl-*N*-(2-hydroxyethyl)amino)pyridine (2.1 g, 13.8 mmol) was dissolved in dry DMF (25 mL) in a 100 mL round-bottom flask. The mixture was stirred under argon in an ice bath, after which sodium hydride (0.906 g, 22.7 mmol) was added. The mixture was stirred at room temperature for 1.5 h before a solution of 4-vinylbenzyl chloride in DMF was added dropwise into the flask. The resulting black mixture was allowed to further stir overnight. After removal of DMF by rotary evaporator, the residue was poured into water and extracted with DCM three times. The combined organic phase was dried over anhydrous Na_2SO_4. After removal of DCM, the crude product was purified by silica gel column chromatography using MeOH/EtOAc (v/v, 2:1) as eluent to yield the product VEMAP as a brown liquid, as confirmed by ^1H NMR spectroscopy (Figure S2B).[2]

Preparation of CHS seed particles (core) and VEMAP particles

CHS seed particles and VEMAP particles were prepared by surfactant-free emulsion polymerization[5]. A typical procedure is as follows: styrene (5 mL), divinylbenzene (0.312 mL), water (50 mL) and a defined amount CHS (or VEMAP) were added into a three-necked flask. The mixture was stirred at 250 rpm by a mechanical stirrer and degassed by argon bubbling for 30 min at room temperature. After heating the mixture to 70 °C, 2,2'-azobis(2-methylpropionamide) dihydrochloride (AIBA, 0.075 g) was dissolved in water (1 mL) and injected into the flask to start the reaction. The reaction time for CHS seed should be well controlled to avoid the aggregation, the reaction was terminated after 4 h and a milky white solution was obtained. To investigate the copolymerization of the CHS

(or VEMAP) and St, non-crosslinked poly(styrene-*co*-CHS)/ poly(styrene-*co*-VEMAP) particles without DVB were prepared. After washing three times with MeOH and drying in oven overnight, the compositions of the particles were determined by ^{1}H NMR (see Figure S3 & S4).

Preparation of CHS/VEMAP CSMs

CHS/VEMAP CSMs particles were prepared by seeded polymerization. Pre-synthesized CHS particles were used as seeds, 22.5 mL CHS particles were mixed with 27.5 mL water, 5 mL styrene, 0.312 mL DVB and relative amount VEMAP. The mixture was degassed by argon bubbling for 30 min before heating to 70 °C. 1 mL aqueous solution containing 0.05 g of AIBA was then added to start the polymerization. In order to obtain (shell-only labelled) core-shell particles for confocal microscopy (Figure S10 & Figure 1B), 10 mg of fluorescein *O*-methacrylate was copolymerized in the presence of styrene, DVB and VEMAP following the rest of the procedure.

Deprotection of CHS/VEMAP CSMs

The thermal deprotection of the particles should be performed with care. For the efficient deprotection of non-crosslinked poly(styrene-*co*-CHS) particles in organic solvent (*e.g.* DMSO, DMF, toluene), the polymer only needs to be heated to 120 °C for 20 min. For the crosslinked particles, they first have to be transferred from water to organic solvent by repeated centrifugation/redispersion cycles (*e.g.* from water to ethanol to organic solvent) before being heated to 120 °C for 3 h. To confirm the deprotection of CHS, non-crosslinked poly(styrene-*co*-CHS) particles was heated to 120 °C for 3 h and evaluated by ^{1}H NMR (see Figure S3B).[4]

Catalyst content test of SSA/VEMAP CSMs

The SSA and VEMAP contents of CSMs were determined by titration. In order to verify the SSA content, specific amounts of VEMAP molecules were mixed with CSMs and used as catalysts for the acid-catalyzed deacetalization serving as indicator to show the degree of neutralization. A typical process is as follows: 0.2 mL SSA/VEMAP CSMs suspension was pre-mixed with specific amounts of VEMAP molecules and 26.3 µL benzaldehyde dimethyl acetal were added in a glass vial. Then the mixture was stirred in a pre-heated oil bath at 90 °C for 2 h. The content of starting reagent **1** and intermediate product **2a** was measured by ^{1}H NMR (see Figure S5). Similarly, to test the VEMAP content, specific amounts of SSA molecules were mixed with CSMs and used as catalysts for the Knoevenagel condensation serving as indicator to show the degree of neutralization. A typical process is as follows: 0.2 mL SSA/VEMAP CSMs suspension pre-mixed with specific amounts of SSA molecules, 26.3 µL benzaldehyde dimethyl acetal and 18.7 µL ethyl cyanoacetate were added in a glass vial. Then the mixture was stirred in a pre-heated oil bath at 90 °C for 6 h. The content of intermediate product **2a** and final product **3** was measured by ^{1}H NMR (see Figure S6).

Catalytic performance test of SSA/VEMAP CSMs

The catalytic performance of the core-shell particles was studied by a deacetalization-Knoevenagel cascade reaction[6–8]. A typical process is as follows: 0.2 mL SSA/VEMAP particle suspension, 26.3 µL benzaldehyde dimethyl acetal and 18.7 µL ethyl cyanoacetate were added in a glass vial. Then the mixture was stirred in a pre-heated oil bath at 90 °C. To test the reaction conversion, the compositions of starting reagent **1**, intermediate product **2a** and the final product **3** were measured by ^{1}H NMR in methanol-*d4*. Compared the integral of the phenylic proton from the final product benzylidene ethyl cyanoacetate (8.35 ppm) with the phenylic proton of the benzaldehyde dimethyl acetal (5.37 ppm) and benzaldehyde (10.00 ppm), the conversions of the starting reagent acetal **1** and the intermediate product **2a** were calculated (see the example in Figure S13).

SUPPORTING INFORMATION

Supporting Figures

Synthesis of the CHS monomer

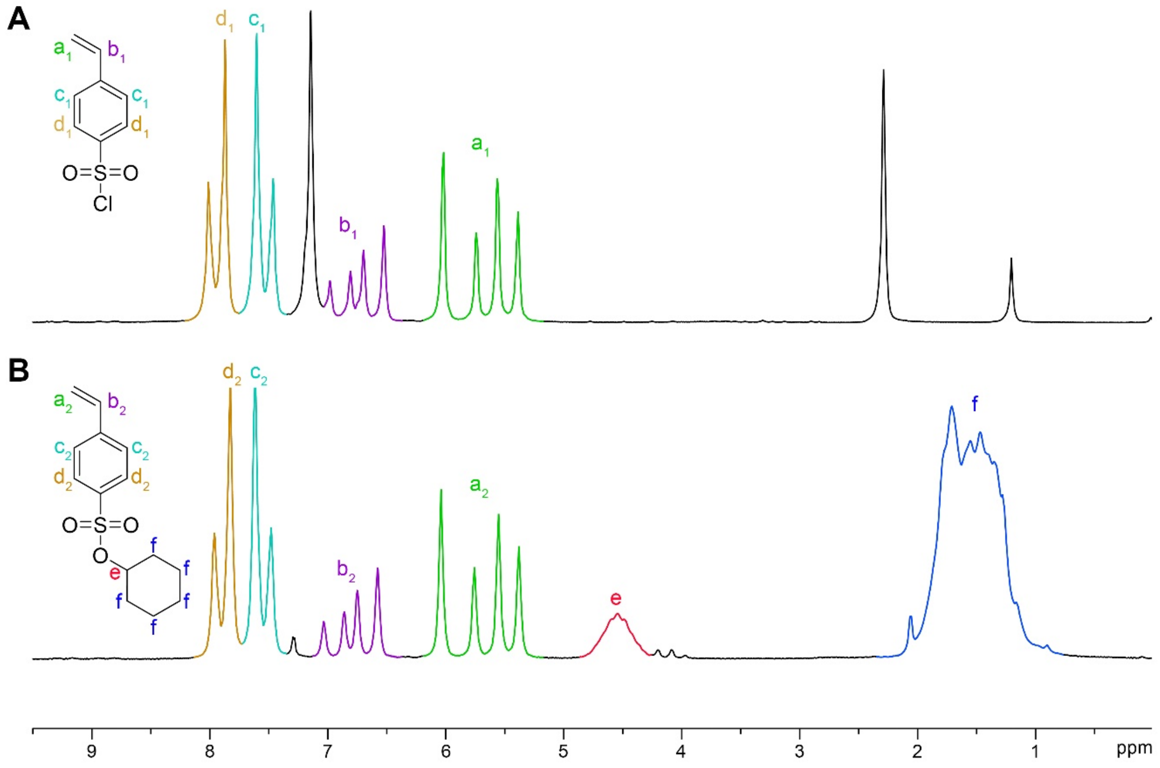

Figure S1. ^{1}H NMR spectra ($CDCl_3$) of the *para*-styrene sulfonyl chloride ***p*SSC** (A) and cyclohexyl-*para*-styrenesulfonate **CHS** (B).

Synthesis of the VEMAP monomer

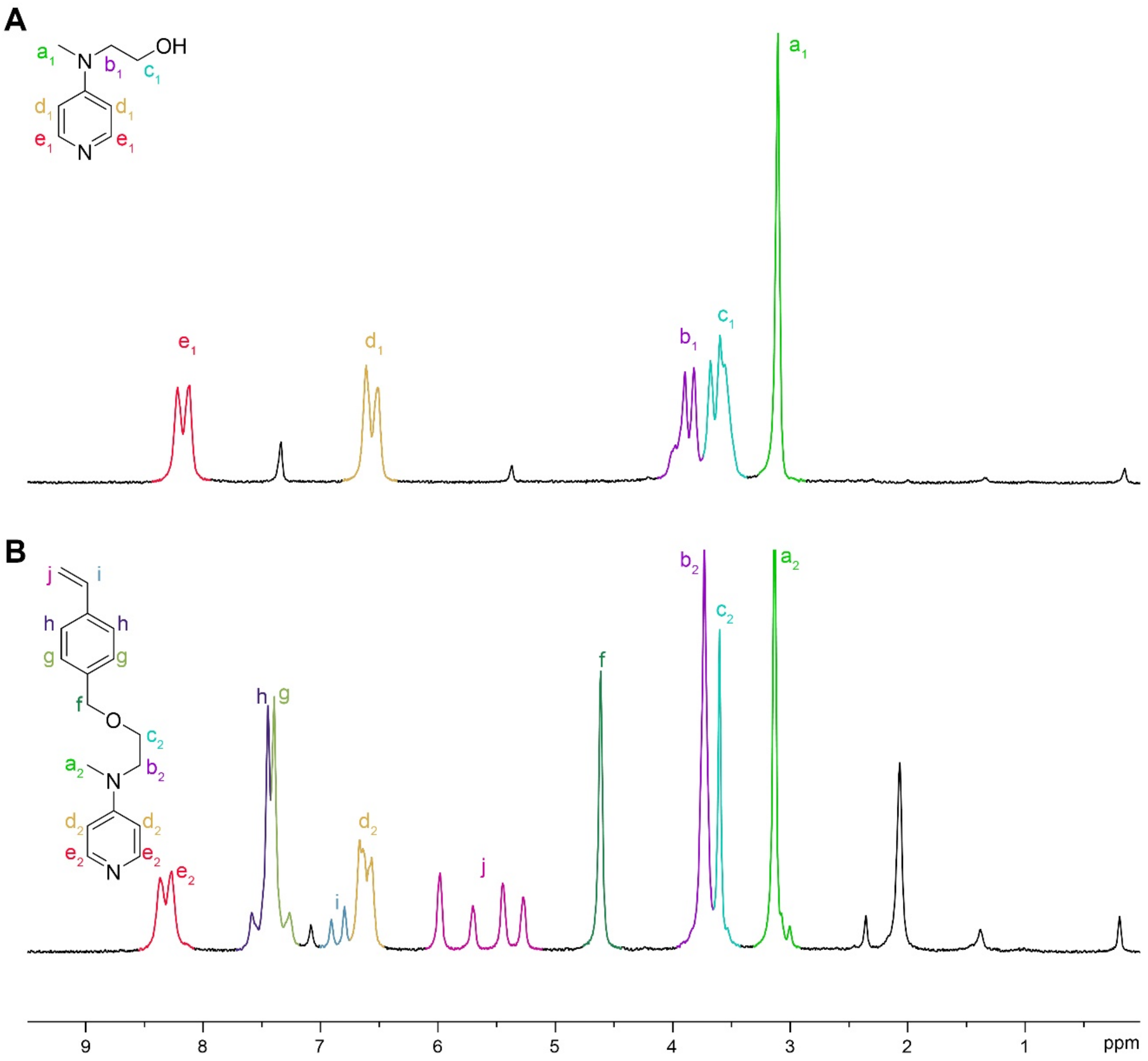

Figure S2. ^1H NMR spectra ($CDCl_3$) of the 4-(*N*-methyl-*N*-(2-hydroxyethyl)amino)pyridine **HEMAP** (A) and 4-*N*-(4-vinylbenzyl)oxyethyl-*N*-methylaminopyridine **VEMAP** (B).

Co-polymerization of St and CHS and the thermo-deprotection of the CHS/St particles

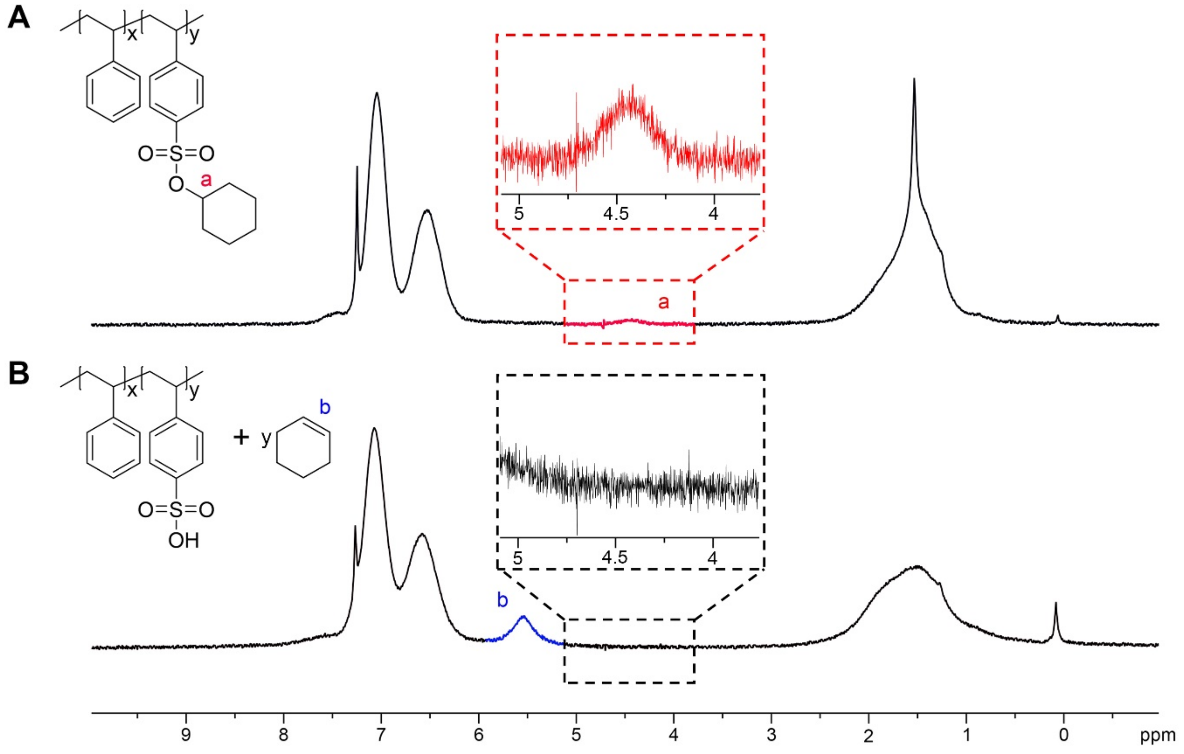

Figure S3. ^{1}H NMR spectra ($CDCl_3$) of the poly(styrene-*co*-CHS) particle (A) and the deprotected poly(styrene-*co*-CHS) particle (poly(styrene-*co*-SSA)) (B).

Co-polymerization of St and VEMAP

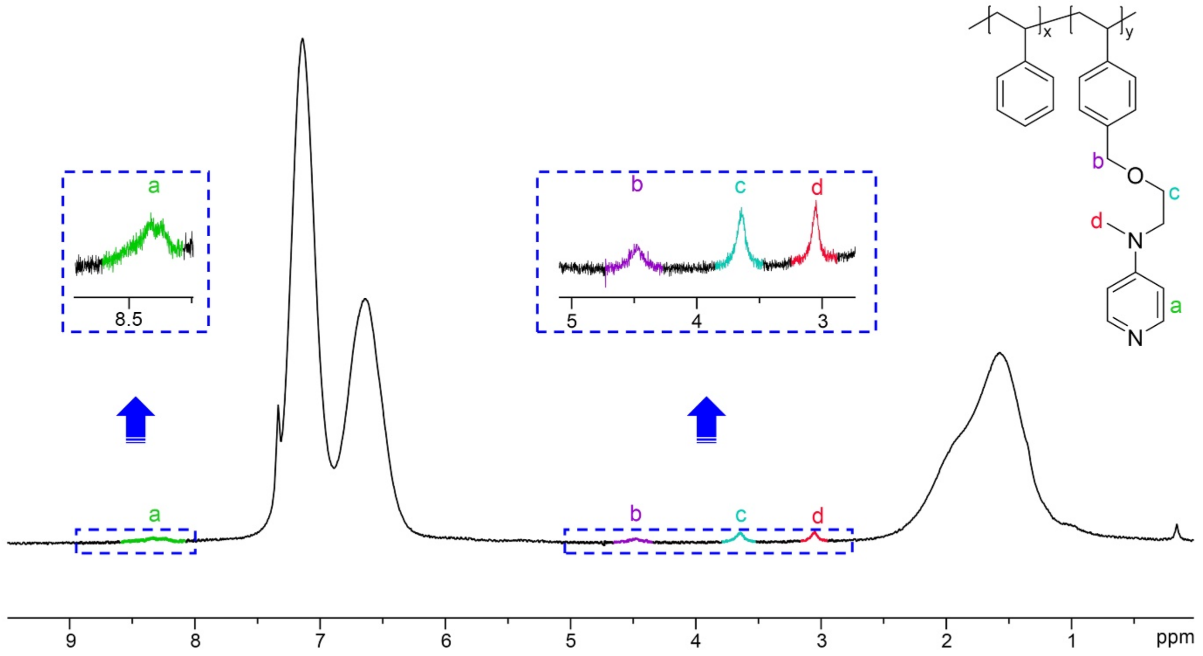

Figure S4. ^{1}H NMR spectrum ($CDCl_3$) of the poly(styrene-*co*-VEMAP) particle.

SSA content test of the CSMs *via* titration.

In order to verify the SSA content inside the CSMs, we employed acid-base titration and used the acid-catalysed deacetalization as indicator. We first mixed the CSMs suspension with specific molar amounts of VEMAP molecules. Afterwards, we performed a series of acid-catalysed deacetalization reactions with the VEMAP-mixed CSMs as catalyst and recorded the conversion by ^{1}H NMR. From Figure S5, pure CSMs without VEMAP molecules added gave nearly full conversion of starting reagent **1** into intermediate product **2a**, and CSMs mixed with VEMAP molecules resulted in lower conversion of starting reagent **1** to intermediate product **2a**. Only a minimal background conversion was found when 0.034 mmol VEMAP was introduced per gram CSMs, which indicated that all SSA catalyst in the CSMs were neutralized by VEMAP molecules. Therefore, we determine the SSA content in the CSMs to 0.034 mmol SSA per gram CSMs.

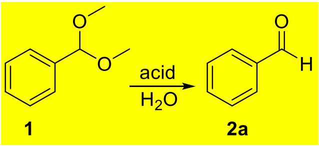

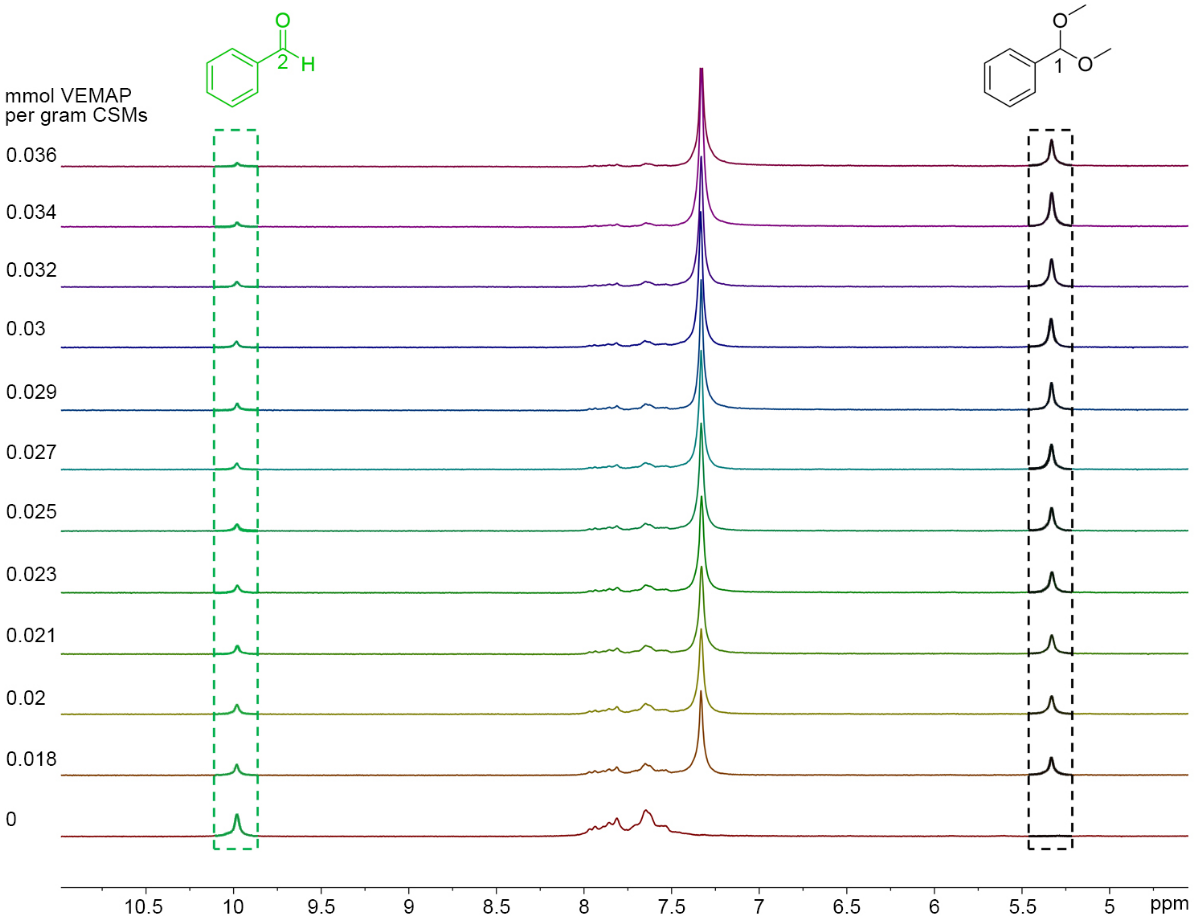

Figure S5. ^{1}H NMR spectra of the benzaldehyde dimethyl acetal hydrolysis using CSMs catalysts with increasing degree of neutralization through added VEMAP molecules. Reaction conditions: 26.3 µL benzaldehyde dimethyl acetal **1**, 0.2 mL CSMs suspension pre-mixed with specific molar amounts of VEMAP molecules per gram of CSMs as indicated in the spectra, 90 °C, 2 h.

VEMAP content test of the CSMs *via* titration.

In order to verify the VEMAP content inside the CSMs, we also employed acid-base titration and used the base-catalysed Knoevenagel condensation as indicator. We first mixed the CSMs suspension with specific amounts of SSA monomer. Afterwards, we performed the base-catalysed Knoevenagel condensation using CSMs catalysts mixed with the SSA molecules and recorded the conversion by ^{1}H NMR. From Figure S6, pure CSMs without SSA molecules gave nearly full conversion of intermediate product **2a** into final product **3**, while CSMs mixed with SSA molecules resulted in lower conversion of intermediate product **2a** to final product **3**. No (or trace) final product **3** was found when 0.094 mmol SSA per gram CSMs was added, which indicated that all VEMAP catalyst in the CSMs was completely neutralized by SSA corresponding to a VEMAP content in the CSMs of 0.094 mmol VEMAP per gram CSMs.

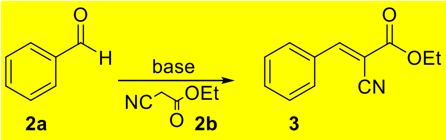

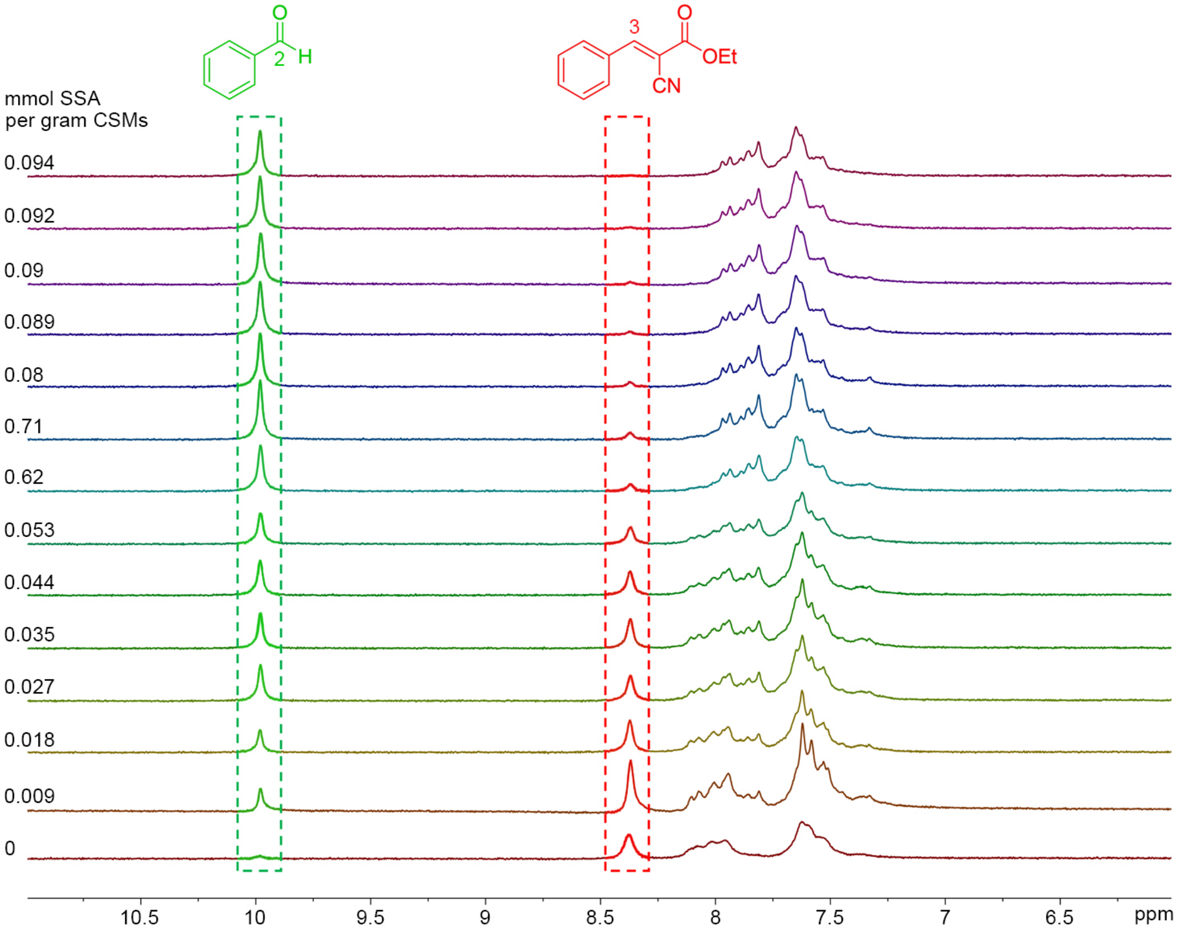

Figure S6. ^{1}H NMR spectra of the Knoevenagel condensation using CSM catalysts with increasing degree of neutralization through the SSA molecules. Reaction conditions: 26.3 µL benzaldehyde dimethyl acetal 1, 18.7 µL ethyl cyanoacetate 2b, and 0.2 mL CSMs suspension pre-mixed with specific amounts of SSA molecules, 90 °C, 6 h;

Influence of initiator for the polymerization of CSMs.

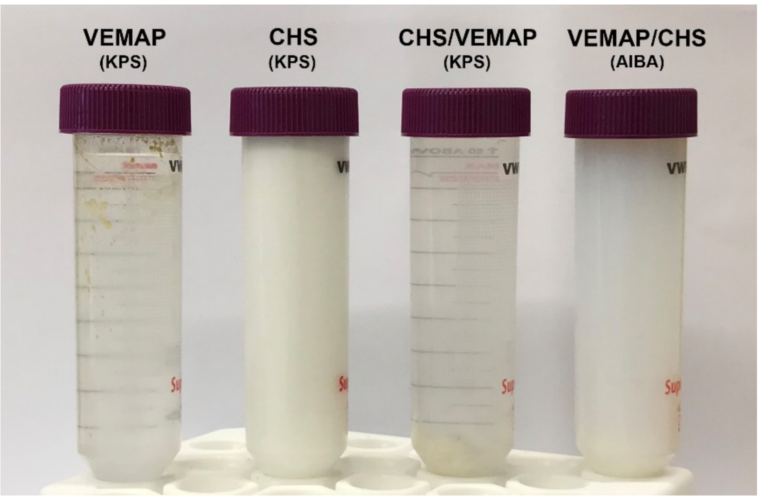

Figure S7. Photograph of centrifuge tubes containing emulsion polymerization residuals, the residuals are VEMAP particles (1st left, KPS as initiator, unstable), CHS particles (2nd left, KPS as initiator, stable), CHS/VEMAP particles (2nd right, CHS particles as seeds and KPS as initiator, aggregated) and VEMAP/CHS particles (1st right, VEMAP particles as seeds and AIBA as initiator, aggregated), respectively.

Influence of polymerization time of CHS seed

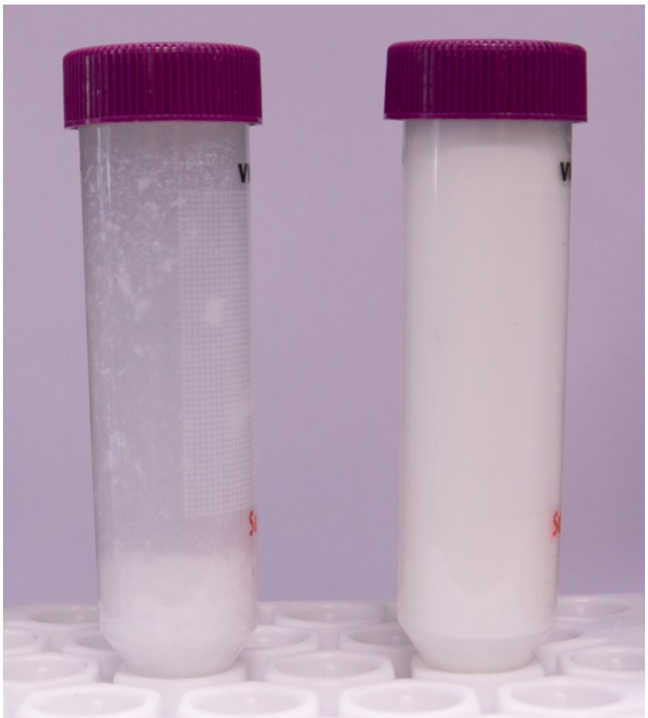

Figure S8. Photograph of centrifuge tubes containing CHS seeds obtained after a reaction time of more than 4 h (left) and a reaction time of 4 h (right). The emulsion becomes unstable if reaction time exceeds 4 h.

Dynamic light scattering measurements

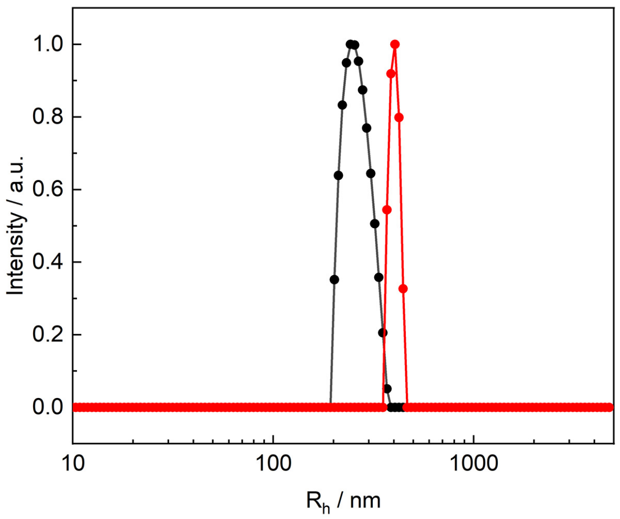

Figure S9. DLS results of the particles size distribution (R_h) of CHS seeds (black curve) and SSA/VEMAP core-shell particles (red curve).

The CLSM overview image of the CSMs.

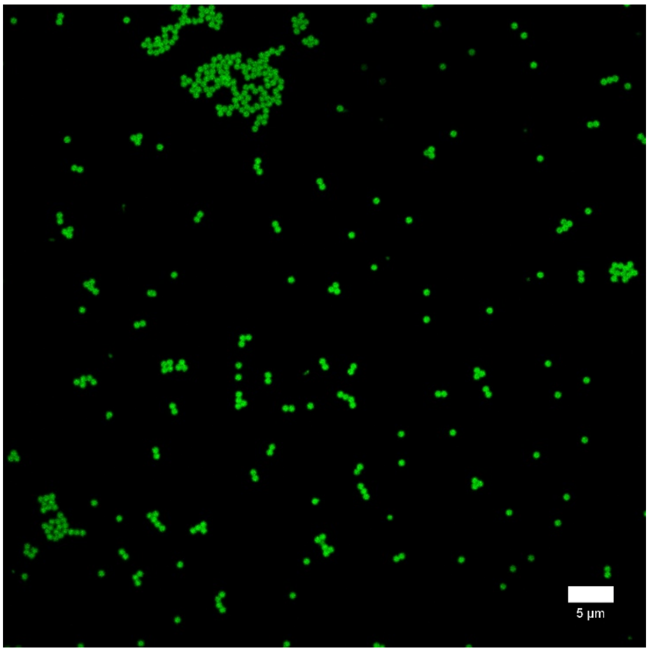

Figure S10. A low-magnification (overview) CLSM image of (shell-only labelled) CSMs.

Reaction kinetics of CSMs in DMSO/H_2O (40:1 V/V).

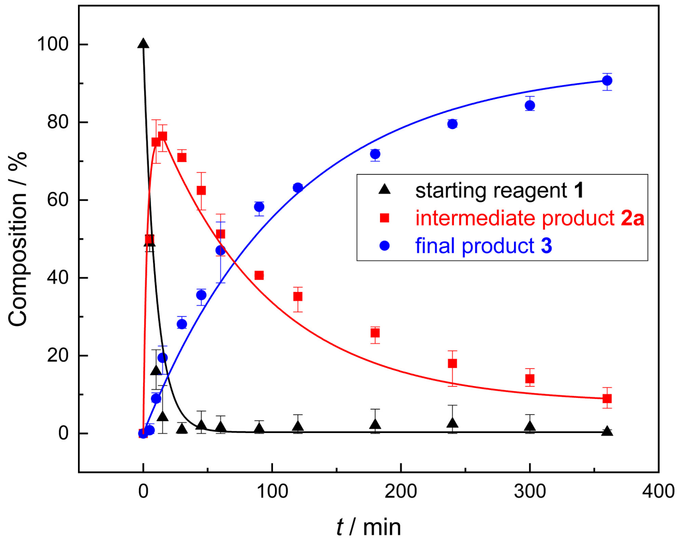

Figure S11. Reaction kinetics of SSA/VEMAP CSMs in DMSO/H_2O (40:1 v/v) followed over time in NMR.

Catalytic performance comparison of CSMs and mixture of individual particles in DMSO/H_2O (40:1 v/v).

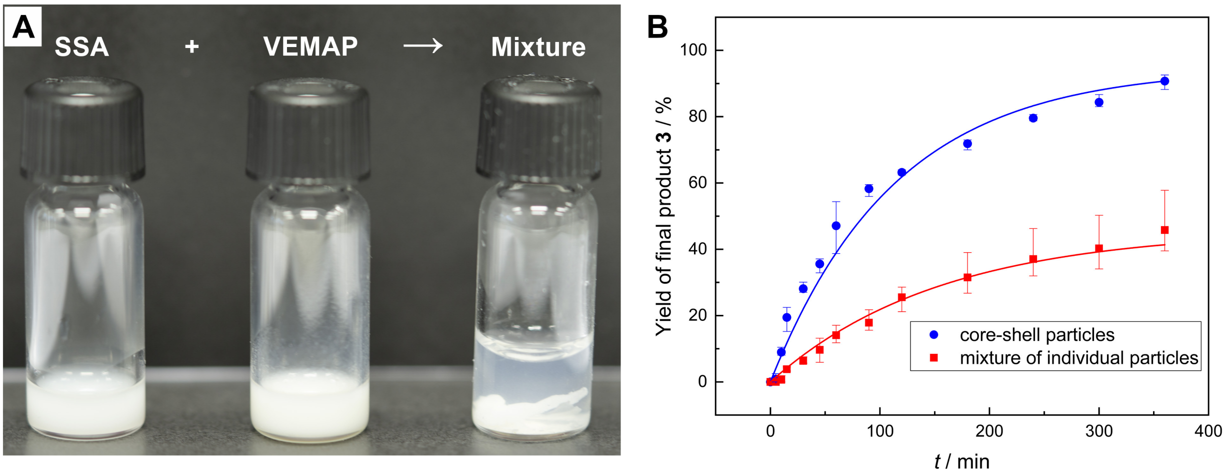

Figure S12. Photograph of SSA particle suspension (left), VEMAP particle suspension (middle) and the mixture of individual particles (right, SSA and VEMAP particles aggregated immediately after mixing) (A); and the reaction profiles of CSMs and the mixture of individual particles for the deacetalization-Knoevenagel cascade reaction (B).

Kinetics of cascade reaction

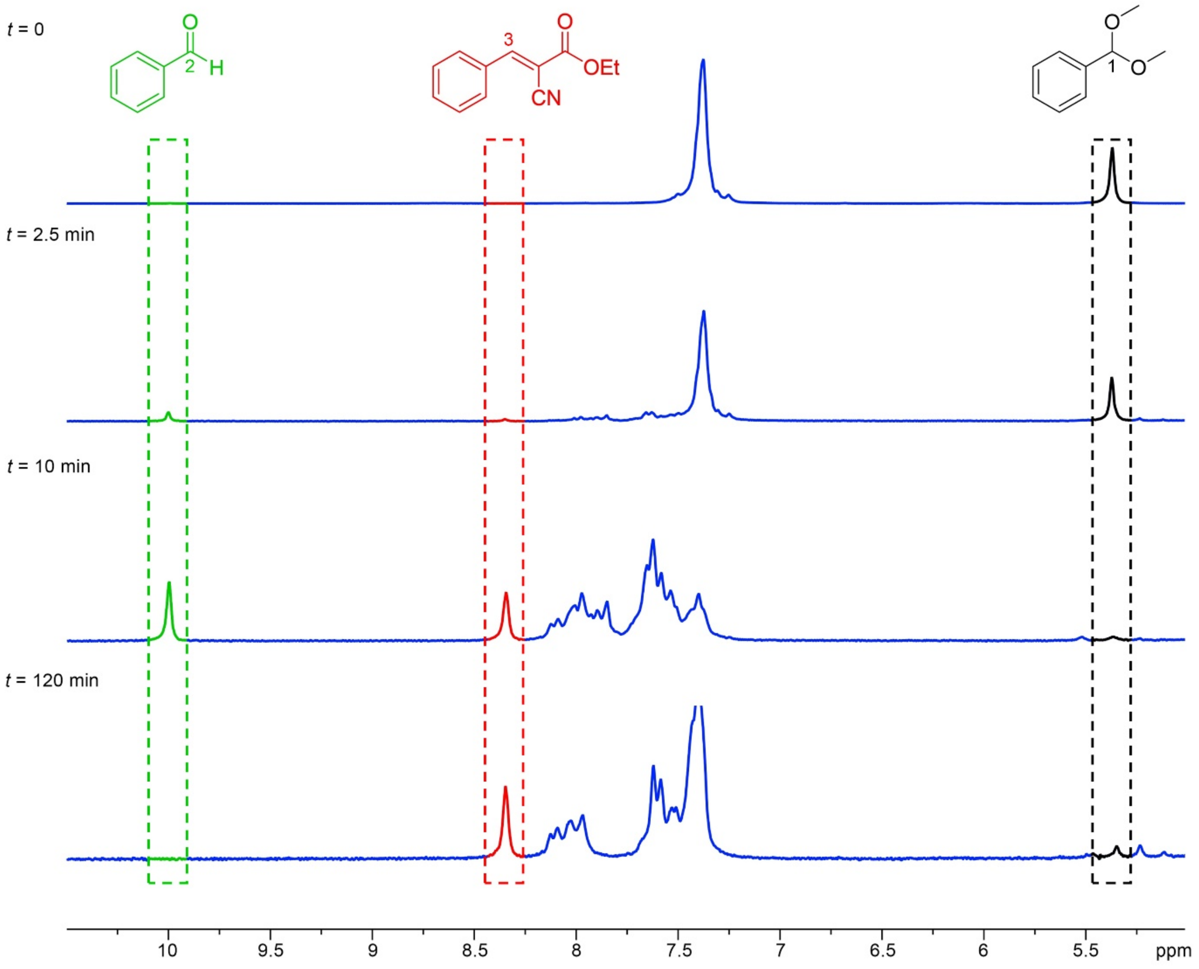

Figure S13. ^{1}H NMR spectra (methanol-*d*4) of the cascade reaction at different times (0 min, 2.5 min, 10 min and 120 min).

SEM image of CSMs after recycling

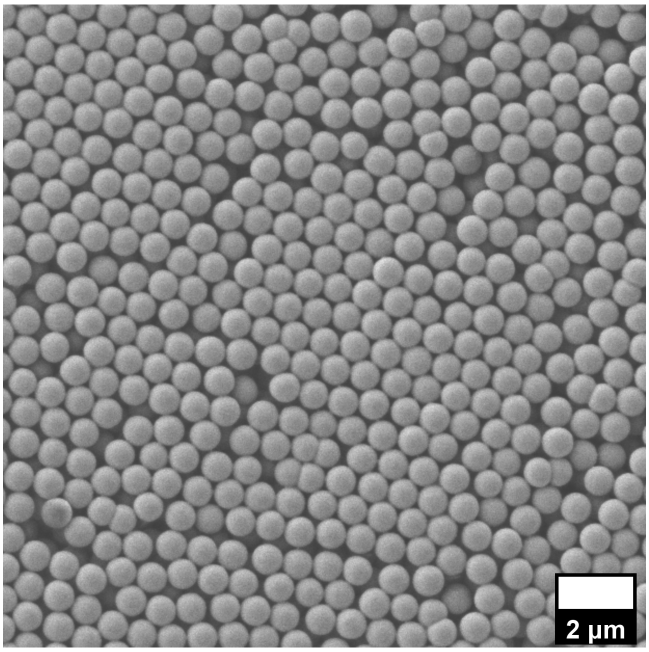

Figure S14. SEM image of CSMs after recycling.

Scale-up potential of surfactant-free emulsion polymerization.

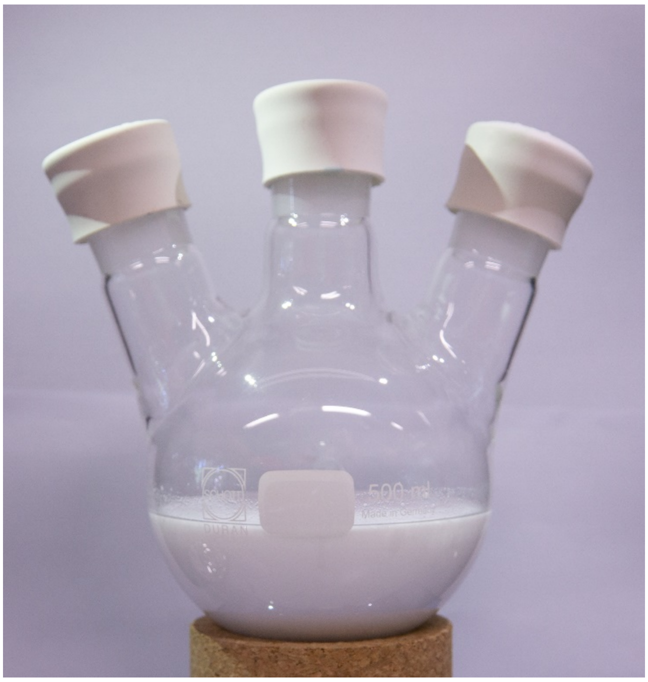

Figure S15. Photograph highlighting the scale-up potential of our core-shell particles (250 mL, 100 g/L).

Scaled-up potential of cascade reaction.

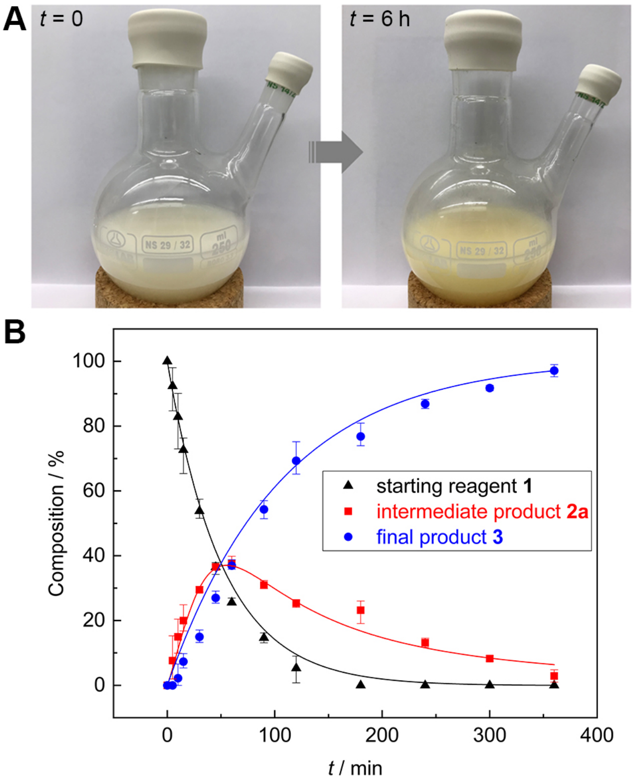

Figure S16. Photographs of flask containing cascade reaction mixture (11.8 mL benzaldehyde dimethyl acetal 1, 9.35 mL ethyl cyanoacetate 2b, 100 mL DMSO containing 940 mg of SSA/VEMAP CSMs and 2.5 mL H2O) before (left) and after reacting for 6 h at 90 °C (right) (A) and the corresponding reaction kinetics of the scaled-up cascade reaction followed over time in NMR (B).

Supporting References

[1] M. Shirai, M. Endo, M. Tsunooka, M. Endo, *Microelectron. Eng.* **2000**, *53*, 475–478.

[2] B. Zhao, X. Jiang, D. Li, X. Jiang, T. G. O'Lenick, B. Li, C. Y. Li, *J. Polym. Sci. Part A Polym. Chem.* **2008**, *46*, 3438–3446.

[3] F. Huber, S. F. Kirsch, *Chem. Eur. J.* **2016**, *22*, 5914–5918.

[4] M. Shirai, J. Nakanishi, M. Tsunooka, T. Matsuo, M. Endo, *J. Photopolym. Sci. Technol.* **1998**, *11*, 641–644.

[5] K. Tauer, R. Deckwer, I. Kühn, C. Schellenberg, *Colloid Polym. Sci.* **1999**, *277*, 607–626.

[6] H. Yang, L. Fu, L. Wei, J. Liang, B. P. Binks, *J. Am. Chem. Soc.* **2015**, *137*, 1362–1371.

[7] N. R. Shiju, A. H. Alberts, S. Khalid, D. R. Brown, G. Rothenberg, *Angew. Chem. Int. Ed.* **2011**, *50*, 9615–9619.

[8] F. Zhang, H. Jiang, X. Li, X. Wu, H. Li, *ACS Catal.* **2014**, *4*, 394–401.

Author Contributions

C.C. conducted all experiments and wrote the manuscript. N.J. assisted in the synthesis of CHS and VEMAP monomers and performed DLS measurements. C.K.W. discussed and wrote the manuscript. C.G. contributed to NMR measurements. R.W. was involved in the manuscript writing process. A.H.G. conceived and designed the experiments and wrote the manuscript. The manuscript was read and approved by all the authors before submission. A.H.G. supervised the project.
